# Archaeal Genome Guardians Give Insights into Eukaryotic DNA Replication and Damage Response Proteins

**DOI:** 10.1155/2014/206735

**Published:** 2014-02-20

**Authors:** David S. Shin, Ashley J. Pratt, John A. Tainer

**Affiliations:** ^1^Life Sciences Division, Lawrence Berkeley National Laboratory, 1 Cyclotron Road, MS 6R2100, Berkeley, CA 94720, USA; ^2^Department of Integrative Structural and Computational Biology, The Scripps Research Institute, 10550 North Torrey Pines Road, MB-4, La Jolla, CA 92037, USA

## Abstract

As the third domain of life, archaea, like the eukarya and bacteria, must have robust DNA replication and repair complexes to ensure genome fidelity. Archaea moreover display a breadth of unique habitats and characteristics, and structural biologists increasingly appreciate these features. As archaea include extremophiles that can withstand diverse environmental stresses, they provide fundamental systems for understanding enzymes and pathways critical to genome integrity and stress responses. Such archaeal extremophiles provide critical data on the periodic table for life as well as on the biochemical, geochemical, and physical limitations to adaptive strategies allowing organisms to thrive under environmental stress relevant to determining the boundaries for life as we know it. Specifically, archaeal enzyme structures have informed the architecture and mechanisms of key DNA repair proteins and complexes. With added abilities to temperature-trap flexible complexes and reveal core domains of transient and dynamic complexes, these structures provide insights into mechanisms of maintaining genome integrity despite extreme environmental stress. The DNA damage response protein structures noted in this review therefore inform the basis for genome integrity in the face of environmental stress, with implications for all domains of life as well as for biomanufacturing, astrobiology, and medicine.

## 1. Introduction

From the ideas of Lamarck, to Darwin and Mendel, to Huxley and those involved in the modern evolutionary synthesis, the accepted views behind the driving force of inheritance and evolution have certainly themselves “evolved” over time. Likewise, how we categorize different life forms has similarly evolved. Before the advent of tools like the microscope, natural intuition would seem to differentiate living things as either plant or animal. In the mid-1800s, Haeckel expanded these categorical divisions of life, which, by the 1960s, eventually grew to 5 “Kingdoms,” allowing the *Monera*, *Protista,* and *Fungi* to be positioned alongside the *Animalia* and *Plantae* [1, 2]. Later, the two-empire system, whose highest level encompassed prokaryotes (“before the kernel,” i.e., lacking a membrane-bound nucleus) and the eukaryotes (containing a “true kernel”), gained attraction as a model to classify life alongside the 5 kingdom system [3]. Beliefs at the time were that these predominantly unicellular organisms that lacked a nucleus must be a less complex predecessor of the more complex eukaryotic cells.

Fast-forwarding to more recent times, the advent of DNA sequencing, along with its subsequent improvements in cost and speed, led to a redefining of the evolutionary divisions of life by providing a shift from phenotypic taxonomy to genotypic computationally aided phylogenetics. Previously, archaea and bacteria were combined as prokaryotes (or monera), based largely upon their similar features (or lack thereof) when compared to eukaryotes: the lack of membrane-bound nuclei and organelles, and being generally unicellular [3]. The sequencing of ribosomal RNA genes eventually led to the separation and clustering of the prokaryotes, which ultimately gave rise to the main, top-level classification systems used today, defining 3 “domains” of life: archaea and bacteria along with the eukarya [4, 5].

For a general understanding of genome integrity, archaea provide master keys to understanding enzymes and pathways critical to stress response and genome integrity, as archaea include many extremophiles that must withstand great environmental stress. At the most basic level, archaea provide critical data for understanding the “periodic table for life.” Notably, all three domains have proteins containing the 21st proteinogenic amino acid selenocysteine, which is one of the few amino acids synthesized in a tRNA-dependent fashion with its specific incorporation directed by the UGA codon, as noted by Mullenbach and others [6–8]. The striking cross domain conservation for use of the otherwise toxic element selenium may reflect life's origins as well as the element's utility. For genome integrity, widely conserved domains and functional structures may likewise reveal not only the original selection for methods to preserve genome integrity but also essential aspects of stress responses for genome maintenance. Here we will highlight informative examples where archaeal genome maintenance protein structures were found to be more similar in organization to eukaryal structures than to bacterial or perhaps provide more insights into how protein structures impact human health, where the first or only structure(s) of a particular protein system was derived from archaea. In all these cases structural results on archaeal proteins are important cornerstones for understanding human homologs involved in disease. Furthermore, as human cell genetics and biological tools improve along with structural results, data from human system and archaeal systems will further complement each other to provide a deeper and more unified understanding as illustrated for FEN1-PCNA, Mre11-Rad50, Rad51, and XPD systems that are among the examples presented here.

## 2. Archaeal Species Speak for Structural Biology

Interestingly, one of the characteristics of many archaeal proteins led to visual support that these “simpler” organisms may be more related to eukarya at certain levels than their bacterial counterparts. The use of proteins isolated from single microbes have filled an impressive number of highly different niches in biotechnological and industrial applications [9]. Likely one of the most desirous common characteristics for these proteins to possess is long-term stability. Organisms with optimal growth temperatures above 80°C are defined as hyperthermophiles [10], the majority of which are classified as archaea [2], and as such contain proteins that are highly stable. Thus, characterization of systems requiring high stability may be facilitated by using genes or proteins isolated from archaea (or bacteria), which natively reside at high temperature and/or pressure [11]. These results may also help efforts to stabilize eukaryotic proteins by designed mutations, such as seen for superoxide dismutase [12]. Relationships of single-site changes and stability have more than academic interest as mutations that destabilize protein frameworks can cause fatal diseases, also as seen for superoxide dismutase [13–15]. Interest in archaeal species and the development of new applications that exploit their enzymes continues to increase. In part, this is due to the appreciation of just how impressive the diverse range of harsh environments in which they inhabit actually appears to be. These “extremophiles” are found in areas not only of high temperature and pressure but also high alkalinity, acidity, salinity, metal content, and even low temperature [9, 11, 16]. In the basic sciences, thermophilic enzymes have been successfully exploited for use in a variety of applications. Archaeal metalloenzymes in general have provided an improved means of understanding and predicting protein metal ion binding sites [17]. Archaea also opened structural doors for understanding the CRISPR system of genetic regulation [18]. Another readily recognized system in molecular biology includes the thermostable DNA polymerases that catalyze DNA synthesis at elevated temperatures during the polymerase chain reaction (PCR). Similarly, in structural biology, enzymes from archaeal thermophiles are frequently characterized, often recombinantly, for a variety of reasons. First, the inherent stability of proteins expressed in cultured cells [19] often yields better-behaved samples *in vitro* at mesophilic temperatures [20]. Second, structural biology such as X-ray crystallography often requires large amounts of highly purified protein, which can often be accomplished by heat denaturation of mesophilic host proteins during purification of recombinant thermophilic proteins [20, 21]. Third, many thermophilic archaeal proteins are significantly closer in amino acid sequence similarity to human proteins than are their thermophilic bacterial counterparts. This aids comparative analyses and inferences into structure-function relationships on the individual atom-, amino acid residue-, domain- and subunit interaction-levels that can be translated into human systems of interest when structures derived from human sources are unavailable. As a consequence of these features, the number of characterized structures derived from archaeal source organisms is rising. Notably, over 3200 such X-ray crystal structures have now been deposited in the protein databank (PDB). Thus, in this postgenomics era, structural biology provides a “seeing is believing” form of support that in many cases, archaea and eukarya together branch off the evolutionary tree from bacteria. Direct structural and three-dimensional computational comparisons of proteins that perform essential basic cellular functions can reveal similarities at the tertiary and quaternary levels between archaeal and eukaryotic proteins [20] and divergence from bacterial proteins. In some instances, archaeal proteins and their structures may be more similar or useful to inform on human proteins of interest than those derived from other eukaryotic model systems like yeast. For example, human and *Pyrococcus furiosus* Rad51 homologs have a similar domain organization, whereas in yeast, there is a significant N-terminal sequence or domain not shared between these homologs. Thus, this information lent to designing a truncated yeast Rad51 construct for crystallization, and due to the stability of the *P. furiosus* protein, a humanized version is being used for inhibitor design [22–26]. This paper will highlight structural insights into several central proteins, enzymes, and complexes involved in basic DNA metabolism, to illustrate key similarities and differences among the three domains.

## 3. DNA Replication and Repair

DNA replication is the basic fundamental process for transferring or copying the “blueprint of life” to budding or dividing cells. Fidelity is required to ensure that errors do not alter the genotype of the cell or are passed on. Death or disease, either at the cellular, organismal, or familial levels, may be a consequence of improper DNA replication. Like other fundamental cellular processes, it would be expected *a priori *that the macromolecules and mechanisms responsible for genome maintenance are conserved in all domains of life. However, early biochemical comparisons of enzymes such as the DNA-dependent RNA polymerases from archaeal *Sulfolobus acidocaldarius* with bacterial and eukaryotic homologs suggested that perhaps archaeal systems involved in nucleic acid metabolism were less similar than bacterial and more similar to and shared properties with eukaryal homologs [27–29]. However, since archaea lack nuclei and typically contain a singular circular genome, this observation appeared counterintuitive. Combining biochemistry with early sequencing efforts determined that archaeal DNA polymerases likewise were seemingly more eukaryotic-like than bacterial polymerases [30, 31]. The sequencing of the first archaeal genome along with other studies further supported the notion that structural and functional aspects of transcription and translation were often similar to those of eukaryotes [32–36].

DNA replication at its heart entails the separation of duplex DNA into two template strands for synthesis of new complementary DNA to give two identical sets of duplex DNA, whereby one set may be allocated to daughter cells. In the initiation phase, DNA is unwound by helicases to provide the template bases and may also be primed by short RNA segments. Because single-stranded DNA (ssDNA) anneals with opposite polarity to form double-stranded DNA (dsDNA), the elongation phase of replicating DNA contains two complementary processes. For the “leading strand” DNA, replication proceeds continuously in the 5′ to 3′ direction along with the replication fork as it is unwound by helicases. For the discontinuously synthesized “lagging strand,” RNA primers are deposited on the template DNA by primase and are extended by another polymerase to generate DNA-RNA Okazaki fragments. RNA primers are later removed, and the gaps on the complementary strand are filled in by polymerases and ligases.

An important principle derived from the double helix but not originally recognized is that the double helix provides the basis not only for DNA replication but also for error-free DNA repair. DNA fidelity within the genome does not depend upon extreme stability of dsDNA but rather on robust DNA repair machinery that extends proofreading by polymerases and responds to all the different forms of DNA damage. For example, damage of DNA bases are repaired by the Base Excision Repair (BER) pathway [37–39], while larger base lesions, crosslinks, or protein-DNA adducts is repaired by the Nucleotide Excision Repair (NER) pathway [40–43]. Other forms of base lesions, such as pyrimidine dimers, apurinic/apyrimidinic (AP) sites, and 8-oxoG, may be bypassed by translesion synthesis (TLS) polymerases [44–46]. Misincorporated DNA bases, or single base insertions or deletions, are repaired by Mismatch Repair (MMR) systems [47, 48]. DNA double-strand breaks (DSBs) that may give rise to threatening gross chromosomal rearrangements are repaired with fidelity by homologous recombination (HR) when possible or by nonhomologous end-joining (NHEJ) in a pinch but with small losses of fidelity [49–51]. Thus, life has evolved such that multiple mechanisms promote fidelity of the genome.

### 3.1. It Starts with a Ring

As mentioned above, replicative polymerases add nucleotides to DNA in the 5′ to 3′ direction, and both strands of dsDNA are used as templates to generate new daughter strands from moving replication forks running in opposite directions. Again, since the two template strands are of opposite polarity, one polymerase (leading) is allowed to run continuously, while the other (lagging) synthesizes DNA discontinuously from constantly added RNA-DNA primers from the primase-Pol*α* complex. Coordinating these efforts in archaea and eukarya is the proliferating nuclear cell antigen (PCNA) protein. PCNA is a multimeric, nonenzymatic scaffold protein that encircles DNA as a ring and is otherwise known as a DNA clamp. In replication, it enhances the activity of the leading and lagging polymerases and also plays a role in Okazaki fragment processing. Besides acting in replication, PCNA also serves as a factor in numerous DNA repair, genome maintenance, and cell cycle processes. This includes DNA repair and recombination pathways such as BER, NER, MMR, and HR [52]. Extensive lists of PCNA protein interaction partners have been noted in reviews [53, 54]. Moreover a variety of posttranslational modifications, including phosphorylation, ubiquitination, and SUMOylation regulate PCNA protein partner interactions in different species.

**Figure 1:**
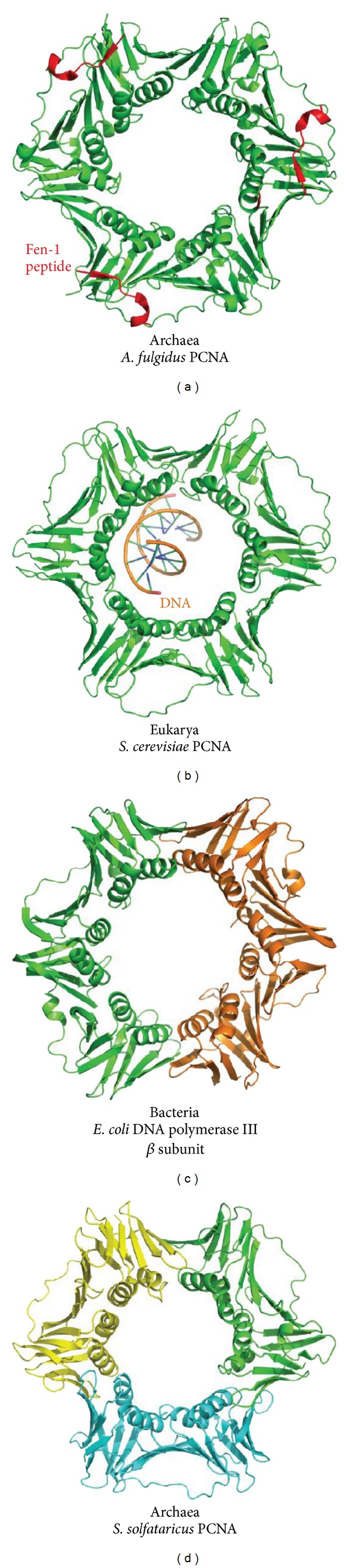
Comparison of PCNAs. The semblance of DNA sliding clamp proteins is made of differing general assemblies. (a) Archaeal *A. fulgidus* PCNA is a trimer composed of three identical subunits, which is the general case for PCNA proteins found in both archaea and eukaryotes. This particular structure has a FEN-1 peptide bound to each subunit (PDB code 1RXZ). PCNA proteins dock other enzymes to bring them into proximity to DNA when their functions are required. (b) The *S. cerevisiae* PCNA shares the general homotrimer assembly with other archaeal and eukaryotic PCNA proteins (PDB code 3K4X). This particular structure was engineered such that a DNA molecule was sequestered within the ring. (c) The *β* subunit of bacterial DNA polymerase III complexes shares the PCNA fold with its archaeal and eukaryotic counterparts. However, the assembly is formed by a homodimer (PDB code 2POL). (d) Archaeal *S. solfataricus* PCNA is unusual as it is assembled as a heterotrimer (PDB code 2HIK). It perhaps evolved this way to dock different enzymes with specificity.

DNA clamp proteins and the enzymes that help them encircle DNA, the DNA “clamp-loaders,” are found in all three domains of life. However, while DNA clamps act as central proteins in a relatively large number of cellular processes, their sequences are generally not conserved. Despite the lack of sequence conservation, their general shapes have preserved features, and the domain and subunit organization of the archaeal and eukaryotic proteins are similar (Figures 1(a) and 1(b)). The majority of archaeal and eukaryotic PCNA proteins are homotrimers [20, 55–57]. The first PCNA structure revealed that each subunit consists of two domains that are topologically similar yet, interestingly, do not share significant sequence identity [57]. A long interdomain connector loop (IDCL) joins the two domains, and an extended *β*-sheet is also formed between subunits. When condensed in head-to-tail fashion into a trimer, the organization is such that an inner ring is formed by 12 *α*-helices, which are flanked by a circumscribing set of 6 *β*-sheets. The organization of fold and assembly of both eukaryotic and archaeal PCNA proteins is shared, again despite lack of sequence similarity. In the bacterium *Escherichia coli*, DNA replicative machinery consists of the large 10-subunit DNA polymerase III (Pol III) holoenzyme. Pol III is divided into the Pol III core, the clamp-loader complex, and the DNA clamp, which is known as the *β*-clamp [56, 58]. With an even greater lack of sequence similarity between the bacterial protein with the eukaryotic and archaeal proteins, coupled with stark differences in protein length, it was likely a surprise to some researchers that the bacterial *β*-clamp shares the overall PCNA ring-shape, including the 12 helix/6 sheet organization [57, 58]. To share the shape in the context of the extended *β*-clamp sequence, the domain and assembly organization differs considerably; it is a homodimer composed of 2 subunits containing 3 domains. In essence, one subunit of a bacterial *β*-clamp resembles one subunit of an archaeal/eukaryotic protein plus one additional domain (Figure 1(c)). Several other PCNA variants have also been discovered in archaea. Crenarcheal homologs, such as that from *Sulfolobus solfataricus*, are heterotrimers composed of subunits PCNA1, PCNA2, and PCNA3 [59, 60] (Figure 1(d)). A recent study suggested the possibility that PCNA from *Sulfolobus tokodaii* forms a heterotetramer from two PCNA2-PCNA3 complexes [61].

Studies have suggested that, in many cases, PCNA stimulates enzymatic activity of partner proteins by influencing their affinity for their respective DNA substrates [62]. To facilitate this type of function with such a large number of proteins (which in turn must be exchanged to meet the needs of reaction steps during pathway progression), some general mechanisms that lend to regulation are expected. As revealed in the *H. sapiens* PCNA:p21 peptide complex structure, followed by others, PCNA uses a conserved binding mode to interact with a number of proteins via the PCNA-interacting protein (PIP) motif [52, 63]. The consensus sequence consists of Q-x-x-L/I/M-x-x-F/Y/W-F/Y/W, where x is a variable amino acid residue. In general, the Gln residue makes contributions to anchor binding of middle residues within this sequence. These middle residues form a short 3_10_-helix and anchor the hydrophobic residues of the motif into a correspondingly hydrophobic pocket on PCNA's surface. The C-terminal end of the peptide then forms *β*-sheet interactions with the IDCL [52, 63, 64]. An additional antiparallel *β*-zipper, analogous to that found in Rad51 [25, 50], is found in some cases [65]. Many enzymes contain this motif at their C-terminus, allowing conformational flexibility and the possibility for more than one protein to bind PCNA at any given time [60, 66]. Again, numerous posttranslational modifications also aid the process of regulating interactions [53, 54]. Recently, advances in solution small-angle X-ray scattering [67, 68] have aided generating experimentally based structures of flexible and multiconformational examples of these systems. This includes visualization of flexibility and the opening and closing of conformational states of PCNA with protein partner Ligase 1 [60], along with regulatory element ubiquitin [69].

### 3.2. Did I Make a Mistake?

Flap endonuclease 1 (Fen1 in archaea or FEN-1 in eukaryotes) plays roles in both DNA replication and repair, often in conjunction with PCNA. During these processes, DNA polymerases synthesize new DNA that displace RNA or damaged DNA creating 5′ single-strand flap structures [70–74]. Similar intermediates are formed during repair processes involving new DNA synthesis, such as long-patch BER (LP-BER). RNaseH may help in the removal of longer RNA stretches but cannot remove the final RNA base [75], and crystal structures and structure-based mutational analysis of RNase HII from *Archaeoglobus fulgidus*, both with and without a bound metal ion, identified it as a molecular ruler and revealed the means whereby type 2 RNase H specifically cleaves the RNA portion of an RNA-DNA/DNA hybrid duplex [76]. Furthermore for short flaps, FEN-1 is the primary structure-specific endonuclease that removes the 5′ ssDNA or RNA flap to produce a single, nicked product that can be sealed by DNA Ligase I [74, 77–79]. FEN-1 can remove 5′ single stranded DNA or RNA flaps from several types of DNA substrates *in vitro* [55, 71, 73, 80–87]. Besides sequence-independent flap endonuclease activity, FEN-1 has other nuclease activities that include 5′ exonuclease activity during recombination and gap-dependent endonuclease (GEN) activity to aid replication fork processes [88–91].

FEN-1 defects are associated with genomic instability and subsequent development of cancer [92–94] and other diseases [95, 96] in eukaryotes. Preventing PCNA-FEN-1 mutations in mice gave rise to defects in RNA primer removal, which was subsequently embryonic lethal [97]. Screening of human cancers for FEN-1 mutations revealed that defects could be identified that affect 5′ exonuclease activity and GEN activity. When one such mutation was transferred to a mouse model, progeny developed autoimmunity and chronic inflammation in addition to cancer predisposition [94]. Therefore the roles of FEN-1 as a structure-specific flap endonuclease, a 5′ exonuclease, and a gap-dependent nuclease have important implications for human health.

The discovery of the first archaeal Fen1 sequences revealed that they had significantly more sequence similarity with their eukaryotic FEN-1 counterparts than with related bacterial sequences [96]. For example, in viruses and bacteria, Fen1 homologs include T4 RNaseH, T5 5′-3′ exonuclease, and the proofreading element of bacterial Polymerase 1. They also were of comparable length to the eukaryotic proteins suggesting they were independent enzymes and not part of other machinery such as in the bacterial case. Two regions that contain elements responsible for nuclease activity, termed the N (N-terminal) and I (intermediate) domains, are predominant areas of homology between these proteins. Other proteins also have similar domains, such as Xeroderma pigmentosum complementation group G (XPG), which is involved in both Xeroderma pigmentosum and Cockayne's syndrome.

**Figure 2:**
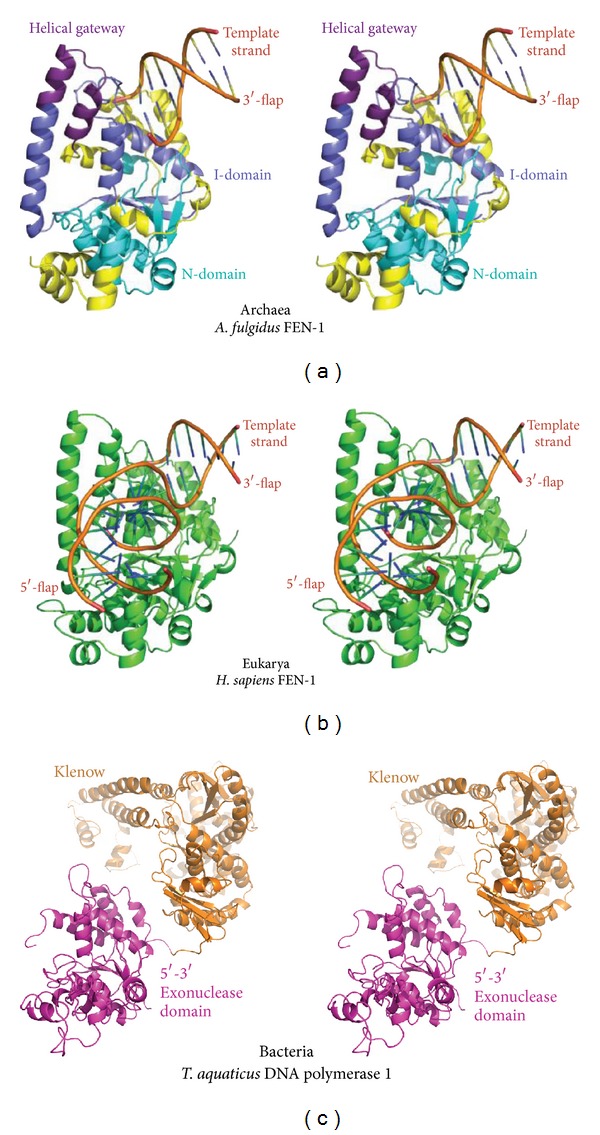
Comparison of FEN structures. Archaeal FEN-1 homologs share structural conservation with eukaryotic FEN1 proteins. (a) Stereoview of archaeal *A. fulgidus* FEN-1 in complex with DNA (PDB code 1RXW). The original conserved N and I regions are shown in light and dark blue, respectively. One strand of the short duplex DNA segment represents a 3′ flap substrate, while the other represents the template strand. The helical gateway segment used to guide DNA is shown in purple. (b) The stereoview of the human FEN1 structure reveals the conservation of tertiary structural fold between archaeal FEN-1 proteins and eukaryotic FEN1 proteins. The more complex double-flap substrate revealed insights into the DNA binding mode and active site chemistry (PDB code 3Q8M). (c) The structurally related “FEN-1” of bacteria is the 5′-3′ exonuclease of DNA polymerase 1 shown in the stereogram in magenta (PDB code 1TAQ). This domain is tethered to the Klenow fragment that carries the DNA polymerase activity.

Due to their stability and ease of purification from heterologous expression systems, the archaeal *P. furiosus *and *Methanococcus jannaschii *Fen1 proteins were the first to be crystallized and structurally characterized [55, 98]. These and other [65] archaeal Fen1 structures revealed that the enzyme is a saddle-shaped, single-domain protein with a ~20 Å deep groove formed from a central seven-stranded *β*-sheet, an antiparallel *β*-ribbon, and two *α*-helical bundles (Figure 2(a)). The C-terminal edge of the *β*-sheet is identified as the substrate-binding region by the presence of catalytically important residues. The two halves of Fen1 are joined by a “helical clamp” or “helical gateway,” which, depending on the set of coordinates, ranges from a flexible unstructured region to a pair of ordered *α*-helices. With additional information from a later DNA-bound human FEN-1 structure, it was found that the protein recognizes DNA 5′ flaps by being able to form a sharp ~100 degree bend with dsDNA on either side (Figure 2(b)). A flap or break is required to bend dsDNA to such a degree at a single phosphodiester site. Binding a 3′ flap causes a ~5 Å shift in the *α*2-*α*3 loop, which creates a “hydrophobic wedge” that packs against the terminal base pair of the DNA. A 3′ flap-binding pocket encloses a single unpaired nucleotide that ensures an eventual product suitable for ligation. FEN-1 also requires the 5′ flap to pass under a cap to enter the helical gateway and the active site. The structure of the bacterial *Thermus aquaticus* Polymerase 1 revealed a relatively conserved fold for the 5′-3′ exonuclease domain that shares homology with the flap endonuclease proteins (Figure 2(c)). The many archaeal results provided a strong foundation for a determination of DNA substrate and product complexes for the human FEN1 as well as FEN1 complexes with PCNA and its repair analogue 9-1-1 that supported and extended the results from archaeal systems [99, 100]. Indeed, the FEN-1 superfamily structure and unpairing mechanism for specificity is broadly conserved with RNA enzymes regulating transcription as well as with replication and repair enzymes such as exonuclease 1, DNA repair protein XPG, endonuclease GEN1, and the 5′-3′-exoribonucleases. Together these enzymes play key roles in many cellular processes such as DNA replication and repair, recombination, transcription, RNA turnover, and RNA interference [101].

With respect to FEN-1 and PCNA functions in DNA replication and repair, questions arise in how the mechanistic steps are regulated. In particular, how are proteins exchanged during these processes, or do multiple proteins bind to PCNA simultaneously? During Okazaki fragment maturation in archaeal replication, work in the *S. solfataricus *system determined that the main proteins involved appear to be PCNA, Fen1, PolB1, and Lig1 [62]. This is analogous to that of PCNA, FEN-1, Pol*δ*, and DNA ligase 1 being the main players for Okazaki fragment processing in eukaryotes [53, 62]. In a homotrimeric PCNA system, it seems logical that binding of one protein partner to a PCNA subunit may either influence the conformations of the unbound PCNA subunits or perhaps sterically exclude other partner proteins, until either DNA conformations or product complex conformations induce a “handoff” to the next protein in the pathway. For the heterotrimeric *S. solfataricus* PCNA, the differing subunits bind their cognate partners as follows: PCNA1:Fen1, PCNA2:PolB1, PCNA3:DNA ligase 1 [59]. Furthermore, data suggests that these interactions may occur simultaneously. *In vitro*, multiple FEN-1 proteins may bind a single eukaryotic PCNA molecule; however, crystallographic information revealed that they did so with different binding modes [66]. Therefore, it is plausible that PCNA may bind different protein partners simultaneously. Moreover, it appears that both inactive “carrier” and “active” conformations may exist in some circumstances when binding a particular protein. It does also appear however that one partner protein may also displace another, as is the case for the DNA clamp loaders and polymerases. Therefore, PCNA's binding interactions appear to be regulated at many levels.

### 3.3. They Dislike U

High temperatures can increase deamination of cytosine resulting in conversion into uracil [102]. Therefore, many archaea have multiple means to control the presence of uracil in DNA and thus suppress possible mutagenic or genotoxic effects. For instance, some archaeal family-B DNA polymerases have a “read-ahead” scanning mechanism employing an N-terminal pocket to detect template-strand uracil and halt the polymerase [103–107]. Additionally, the heterodimeric euryarchaeal family-D polymerases may also possess a uracil detection-response system, though likely through a distinct mechanism [108]. Archaea can also contain dUTPases [109] including DCD-DUT, which converts dUTP to dUMP to prevent misincorporation of dUTP into DNA and is exemplified by a jelly-roll fold with two helices and a *β*-arm [110]. Other conserved uracil repair activities include steps leading to BER. Some archaea possess enzymes such as the helix-hairpin-helix folded uracil-DNA glycosylases (UDG) similar to the MIG/EndoIII enzymes [111, 112], and UDGs from other superfamilies are also present in archaea, as discussed below.

**Figure 3:**
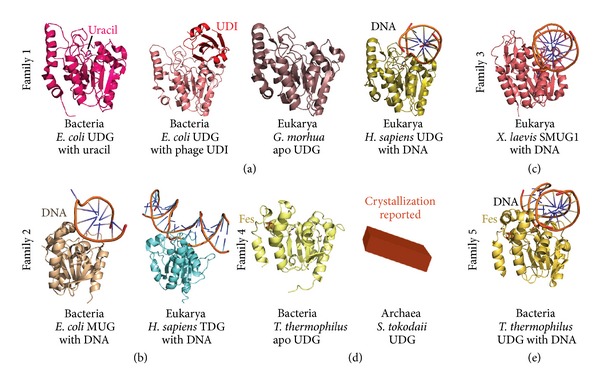
Representative structures of UDGs from families 1–5. (a) Family 1: bacterial UDG monomer with uracil from *E. coli *(PDB code 1FLZ) and* E. coli* UDG (PDB code 1UUG) bound to a *Bacillus* phage inhibitor (UDI). Eukaryotic UDG structures exemplified by apo *G. morhua* UDG (PDB code 1OKB) and DNA-bound human UDG (PDB code 1EMH). (b) Family 2: *E. coli *MUG in complex with DNA (PDB code 1MWJ); human TDG (PDB code 2RBA). (c) Family 3: *X. laevis* SMUG1 in complex with DNA (PDB code 1OE4). (d) Family 4: bacterial UDG (with Fe-S) from *T. thermophilus *UDG (PDB code 1 UI0). Determination of the X-ray structure from *S. tokodaii* will shed light on archaeal UDG homologs. (e) Family 5: *T. thermophilus *UDG (with Fe-S) bound to DNA (PDB code 2DEM). Archaea contain homologs from families 2, 4, and 5.

As a first step in the BER pathway, glycosylases must target incorrect base lesions and cleave the bond between the base and deoxyribose sugar in DNA, creating an apurinic/apyrimidinic (AP) site. Following this step, the actions of conserved AP endonucleases, polymerases, and ligases finalize the repairs [113]. Of the BER-triggering glycosylases, the UDG superfamily is a well-studied example specific to removal of uracil [114]. Within this superfamily, 6 families have been classified [115]. Family 1 is comprised of the uracil DNA N-glycosylases (UDGs/UNGs) [116] and related homologs whose substrates include ssDNA and dsDNA (Figure 3). These enzymes are found in bacteria and eukaryotes, and in humans; the UNG2 protein is involved in somatic hypermutation for immunoglobulin gene diversification in the immune system [117]. Family 2 includes mismatch-specific uracil-DNA glycosylases (MUGs) [118] and thymine-DNA glycosylases (TDGs) [112, 119]; family 3 (mostly eukaryotes and some bacteria) include the single-strand-selective monofunctional uracil DNA-glycosylases (SMUGs) [120]; families 4 [121] and 5 [122] have distinct specificities [123, 124] and contain an Fe-S cluster specific to thermophiles (bacteria and archaea); family 6 are hypoxanthine-DNA glycosylases [125] found in all domains. Thus, of these, only the first 5 contain UDG activity [125]. Archaea appear to utilize UDGs from families 4, 5, and 6 and sometimes 2 [125, 126].

Structures from these UDG family members are typically characterized by a *β*-sheet bordered by *α*-helices (the *α*/*β*/*α* sandwich) and contain a pocket that positions the uracil for cleavage (see Figure 3). Structures of family 4 UDGs also reveal a similar fold with the Fe-S cluster adjacent to the active site. Interestingly, despite a common evolutionary ancestor and fold for family 1–5 UDGs, divergence has been observed at the sequence level and manifests in part via active site differences [126, 127]. Steric features help to recognize uracil via hydrogen bonding, bending of DNA and nucleotide flipping [128]. Two active site motifs are variable between the UDGs and contribute to their subtle mechanistic distinctions [125]. Recently, crystallization of the first archaeal (family 4) UDG from *S. tokodaii *was reported [129]. When this structure is finalized, it will be fascinating to compare the structural determinants of this enzyme with those known from bacterial and eukaryotic UDGs. More generally, variation and conservation of BER enzymes from archaea to humans provide a deeper understanding of strategies to remove or reverse base damage. For example, N1-methyladenine (m1A) and N3-methylcytosine (m3C) are major toxic and mutagenic lesions induced by alkylation in single-stranded DNA. In bacteria and eukaryotes, m1A and m3C are repaired by AlkB-mediated or AlkB-like (ABH) oxidative demethylation [130, 131]. Yet, no AlkB homologues have been identified in Archaea, and m1A and m3C are repaired by the AfAlkA base excision repair glycosylase of *A. fulgidus*, suggesting a different repair mechanism for these lesions in the third domain of life [132].

### 3.4. Unwind or Move On

Large DNA lesions caused by chemicals or UV radiation, such as thymine dimers, threaten genomic fidelity in all three domains of life. Archaea, like bacteria and eukarya, utilize NER to repair such DNA lesions [40]. During NER, either enacted globally or during transcription, damage must be recognized, dsDNA must be unwound and the lesion bracketed with incisions before the damaged stretch of ssDNA can be excised and resynthesized. In contrast with other DNA repair mechanisms, no significant homology exists between the NER machinery in bacteria and eukarya. Bacteria perform NER using a complex of Uvr proteins (UvrABCD) [40, 133], whereas eukaryotes use the multicomponent transcriptional and repair factor TFIIH in addition to other proteins [134]. Archaeal organisms lack many components of eukaryotic TFIIH, but sometimes encode homologs of the bacterial Uvr proteins. Exceptions to this trend are the Xeroderma pigmentosum complementation group D protein, XPD (also known as ERCC2 or Rad3), and Xeroderma pigmentosum complementation group B protein, XPB [135], which both form part of TFIIH in eukaryotes and are encoded by many archaea. XPD and XPB also help regulate general transcriptional as part of the TFIIH complex [136–138].

In both eukarya and archaea, XPD functions as an ATP-driven DNA helicase recruited to unwind dsDNA near the lesion-site for NER. XPD is superfamily 2 (SF2) helicase that contains a pair of Rad51/RecA-like helicase domains (HD1 and HD2), is characterized by the insertion of Arch and Fe-S domains [139] into HD1, and functions as a 5′-3′ helicase. In eukarya, XPD also helps dictate cell cycle progression via cyclin-dependent kinase activating kinase (CAK) interactions [136] and, in humans, the Arch domain may represent a recruitment platform for CAK to TFIIH [140]. The Fe-S domain was proposed to recognize the DNA (at the dsDNA-ssDNA junction) and place the enzyme in an appropriate position for unwinding [141]. The Fe-S domain is also likely to play an important role in 5′-3′ processing, since all Fe-S containing helicases characterized to date operate with 5′-3′ polarity [142]. Furthermore, XPD may serve to verify DNA damage, as yeast Rad3 [143, 144] and *F. acidarmanus* XPD (FaXPD) appear to stall at damaged sites. For FaXPD, this abortion of helicase activity was accompanied by stimulation of ATPase activity [144]. Thus, the damage-specific stalling of XPD was recently described as a central decision point in the NER reaction [145].

**Figure 4:**
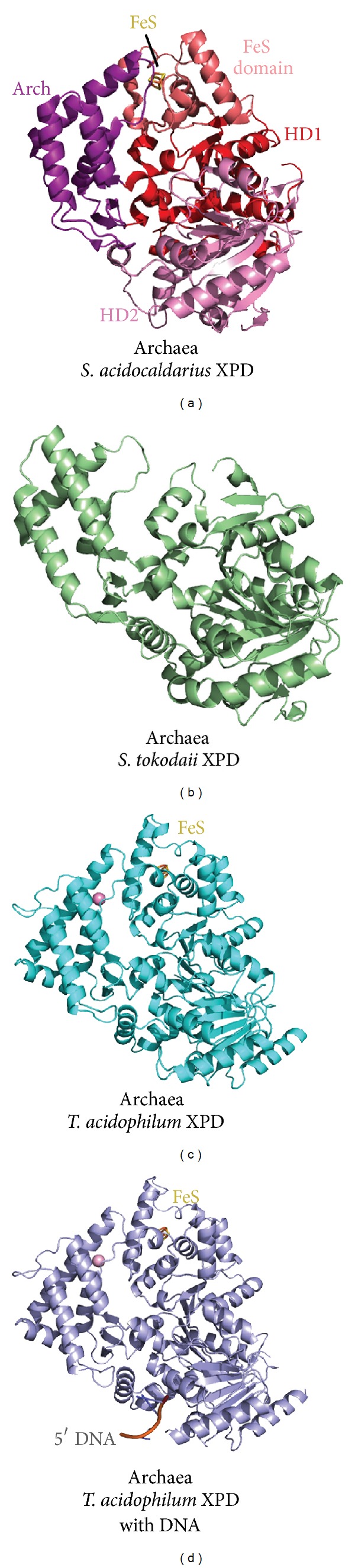
Insights into XPD from archaeal structures. Structures of XPDs from *S. acidocaldarius *(PDB code 3CRV), *S. tokodaii* (PDB code 2VL7), and *T. acidophilum* XPD with (PDB code 4A15) and without DNA (PDB code 2VSF) reveal Arch, Fe-S, HD1, and HD2 domains forming a seat-shaped molecule. Notably, despite similar architecture, cleft pore size between Arch and Fe-S domains varies, suggesting flexibility in this region. DNA orientation is shown for* T. acidophilum *XPD, and a calcium ion is shown as a pink sphere.

Consistent with this critical role, dysfunctions in the NER pathway cause a UV-hypersensitive phenotype in organisms such as humans and yeast, and moreover, deletion of XPD is embryonic lethal in mice [146]. In humans, point mutations in XPD produce three different diseases: xeroderma pigmentosum (XP), Cockayne syndrome with XP (XP/CS), and trichothiodystrophy (TTC) [42]. Structural information has been crucial to elucidating the molecular determinants of XPD mutations. Whereas human XPD has not proved amenable to structural studies, a number of archaeal XPD structures have been solved (Figure 4). The structures of XPD from *S. acidocaldarius *(SaXPD) [147] and *S. tokodaii* (StXPD) [148] have been solved in the absence of DNA, while XPD structures *from T. acidophilum* (TaXPD) have been solved in the presence [149] and absence of DNA [150]. These structures have allowed investigators to rationalize how sequence-related XPD mutants can cause different diseases: ATP and DNA binding mutations give rise to XP; flexibility and conformational mutations give rise to XP/CS; and framework destabilizing mutations give rise to TTD [147, 148]. The structure of TaXPD bound to a 4 nucleotide DNA above motor domain 2 [150] plus spectroscopic results on XPD with bound DNA [151] helped define the path of the translocating DNA through the pore region of the protein created by the arch and FeS domains (Figure 4), as well as elucidating how XPD-like helicases operate in the 5′-3′ direction. Rather than reorienting the DNA, XPD helicases grip it in the same orientation as the 3′-5′ SF2 helicases but process the DNA in the opposite direction. These structures have also helped researchers develop models of DNA-damage detection based on charge transfer (or electron tunneling) between the Fe-S domain and the DNA [142, 152]. Thus, archaeal XPD structures have been critical in helping elucidate more general insights into NER. Indeed, archaeal protein structures have informed the activities of other ATP-driven motors such as the secretion ATPase superfamily [153].

Furthermore, some archaea contain alkyltransferase-like (ATL) proteins, whose protein-DNA complexes can switch base damage to the NER pathway. ATLs share functional motifs with the cancer chemotherapy target O(6)-alkylguanine-DNA alkyltransferase (AGT) and paradoxically protect cells from the biological effects of DNA alkylation damage, despite lacking the reactive cysteine and alkyltransferase activity of AGT. Structural results on the ATL from *Schizosaccharomyces pombe* without and with damaged DNA containing endogenous lesions revealed nonenzymatic DNA nucleotide flipping plus increased DNA distortion and binding pocket size compared to AGT. Analysis of lesion-binding site conservation identified ATLs in sea anemone and ancestral archaea, indicating that ATL interactions are ancestral to present-day repair pathways in all domains of life [154]. Genetic connections to mammalian XPG (also known as ERCC5) and biochemical interactions with *E. coli* UvrA and UvrC combined with structural results reveal that ATLs sculpt alkylated DNA to create a genetic and structural intersection of base damage processing with nucleotide excision repair. Such sculpting of DNA to create cross-talk among different DNA repair pathways may prove to be a general strategy to regulate DNA damage response networks [155].

### 3.5. Staying Together

DNA double strand breaks (DSBs) are a particularly threatening type of DNA damage, posing a risk of genetic information loss. DSBs can occur as a result of DNA replication and repair events or from extrinsic factors or toxins. Various forms of DSB repair exist, and several key double strand break (DSB) repair players are conserved in all domains of life. The repair of DSB typically utilizes one of two major pathways: homologous recombination (HR) or nonhomologous end-joining (NHEJ), but in some instances microhomology-mediated end-joining may be used [156]. In all domains, a core complex termed MR (for Mre11 and Rad50) plays key roles in detecting and repairing DSBs. In eukaryotes a third accessory protein (Nbs1 or Xrs2) is present to form MRN or MRX complexes, respectively.

**Figure 5:**
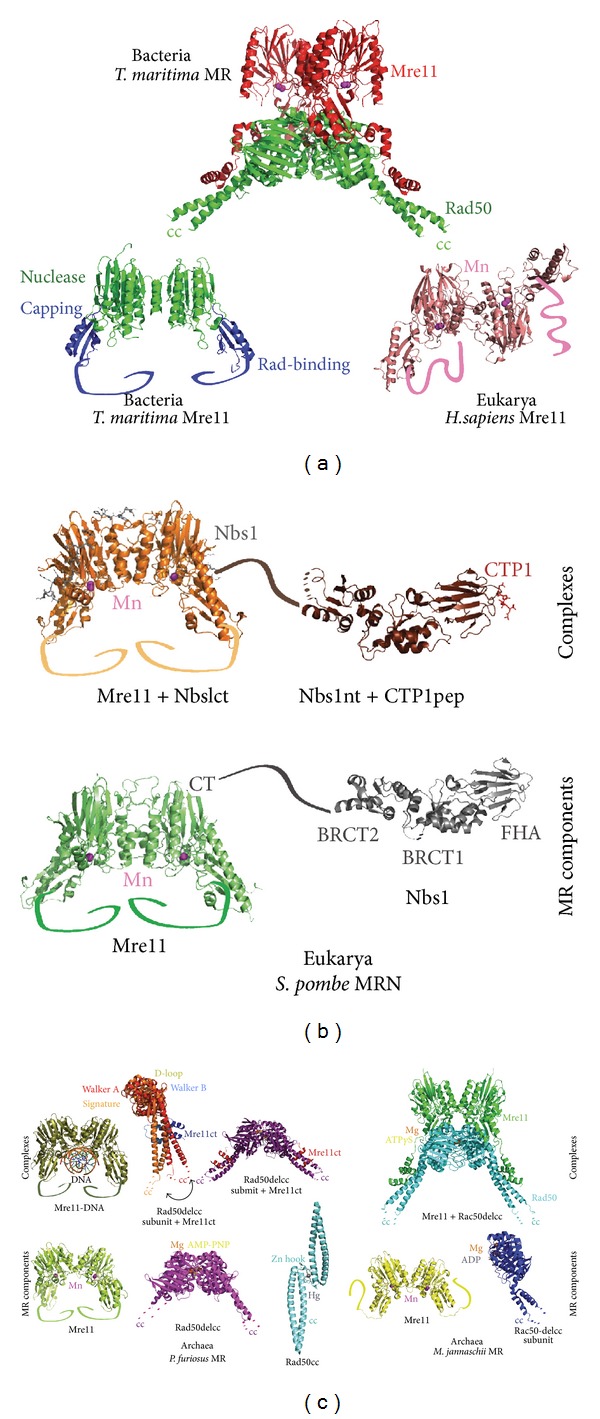
Conservation of MR family members in three domains. (a) Structures of bacterial homologs have elucidated domain organization of MR components. The bacterial Mre11 dimer, exemplified by the *T. maritima *homolog (PDB code 2Q8U), contains the larger, N-terminal nuclease domains (green), the adjacent capping domains, and Rad-family member binding domains (not structurally defined for TmMre11). The Rad50 portion of the Mre11-Rad50 complex (PDB code 3THO) in *T. maritima* is comprised of a curved, globular domain of two lobes with intervening coiled coils (lower regions). (b) Human and yeast MRN structures reveal eukaryote-specific features. The subunit orientation of human Mre11 dimer (PDB 3T1I) is substantially rotated, as compared to other known homolog structures. Mre11 and Nbs1 complexes exemplified by *S. pombe *(PDB codes 4FCX (SpMre11); 4FBW (SpMre11 with SpNbs1 C-terminal region); 3HUE (C-terminally truncated SpNbs1); 3HUF (SpNbs1 in complex with CTP1 peptide)) have revealed key regulatory interactions including the binding site of the Nbs1 C-terminal tail on Mre11 and the FHA domain interaction of Nbs1 with a phosphopeptide of CTP1. (c) Insights from archaeal MR components and complexes. Mre11 structures from *P. furiosus* (PDB codes I117 (PfMre11); 3DSC (PfMre11 with DNA); 3QKT (PfRad50 core); 3QKU (PfRad50 core with PfMre11 C-termini); 3QKS, 3QKR (PfRad50 subunits with PfMre11 C-termini); 1L8D (PfRad50 coiled coil and Zn hook)) have revealed DNA binding and partner interaction sites key to MR assembly. Likewise, complementary *M. jannaschii *structures have confirmed key architecture and interaction sites between MjMre11 and MjRad50 (PDB codes 3AUZ (MjMre11); 3AUX (MjRad50 core); 3AV0(MjRad50 core with MjMre11)).

The first archaeal structures [157–159] combined with recent structural insights have been especially powerful in illuminating our understanding of MR architecture and mechanism, in particular in *P. furiosus* [160, 161], *M. jannaschii *[162], and* T. maritima *[163, 164] (Figure 5). The arch-shaped Mre11 homodimer is assembled by interactions of two manganese-containing nuclease domains, which are each flanked by nuclease capping domains controlling active site access [158] (together comprising the phosphodiesterase domain) for DNA ends [161] and is then trailed by C-terminal Rad50-binding domains often not included in crystallographic structures. Dimer formation is required for stable DNA binding in the cleft between subunits but not endonuclease activity [161]. Rad50 is a dumbbell-shaped ATP-binding cassette protein containing a conserved signature motif [165] with joined ends connected by 600–900 amino acids of coiled coil linker [159] containing a zinc hook mediating complex bridging [157]. The two termini form a bowl-shaped globular domain containing two lobes with a signature motif, N-terminal Walker A and C-terminal Walker B motifs, magnesium ions, and several key loops required for Rad50 catalysis [159]. Upon ATP and Mg^2+^ binding, the MR complex moves from a wing-shaped heterotetramer to a globular structure and as such acts as an ATP-stimulated nuclease to degrade DNA ends and bridge them in repair and recombination [157, 166]. Although a precise mechanism for ATP-coupled catalysis is currently being resolved, recent insights from archaeal MR complexes have been crucial in efforts toward this goal [160, 162] (Figure 5).

Signaling interactions by eukaryotic MRN complexes have also been informed by structural studies on eukaryotic homologs [167]. Structures of Nbs1 domains alone [161, 168, 169] and bound to a CTP1 peptide [161] or Mre11 [167, 170] have illuminated our knowledge of the eukaryotic-specific components. Moreover, human Mre11 contains a distinct orientation of the dimer heads [170]. In metazoans, these complexes are linked to the cell cycle, telomere maintenance, and activation of ATM kinase. Overall the utility of the complexes are underscored by observations that mutations in MR components lead to disease or severe phenotypes [171]. Nonetheless, archaea may also have domain-specific interacting partners, for instance, the DNA-associated MlaA (or HerA) ATPase that may work with MR in processing (or restarting) stalled replication forks or Holliday junctions in hyperthermophilic archaea [172]. The intricate functions of the MR complex await further characterization.

### 3.6. Protection

Homologous recombination (HR) is regarded as an ancient essential DNA metabolism system [51] that plays important roles in the repair of DNA DSBs from exogenous agents, replication associated repair of DSBs, gene rearrangement, mitosis, and meiosis [50, 51, 173]. While the basic process of HR is conserved among the three domains of life, only the central enzymes are conserved. Many other enzyme factors, usually dubbed mediators in the recombination field, aid steps of the reactions and also perform key signaling functions in specific species.

**Figure 6:**
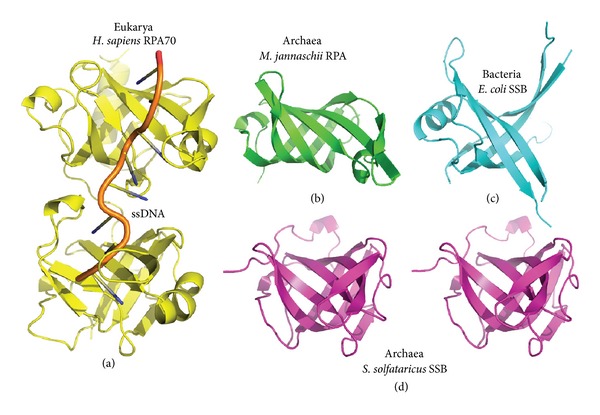
Single-stranded DNA binding proteins. Bacterial members of this class of protein are generally termed SSBs, which stands for single-stranded DNA binding proteins, whereas, originally, eukaryotic versions were called replication protein A (RPA). Archaeal single-stranded DNA binding proteins are generally split between the euryarchaeal RPA proteins and the crenarchaeal SSB proteins. The DNA binding elements of these single-stranded DNA binding proteins are oligosaccharide/oligonucleotide binding (OB) folds. (a) Two tandem OB folds representing residues 181–422 from the largest subunit of human replication protein A, RPA70 (PDB code 1JMC), reveal the binding mode to ssDNA. (b) Example of the OB fold from the euryarchaeal *M. jannaschii *RPA structure (PDB code 3DM3 chain A). The structure is in the same orientation as the top domain in (a). (c) An example of a bacterial SSB domain from *E. coli*. (PDB code 1SRU chain A). (d) The characterization of a crenarchaeal SSB protein from *S. solfataricus*, whose OB fold (stereoview shown in same orientation as the bottom domain in (a), PDB code 1O7I chain A) resembles that of eukaryal RPA. Interestingly, its overall domain organization is more similar to bacterial SSBs, and this led to the discovery of additional single-stranded DNA binding proteins in humans.

HR begins with end resection to form 3′ ssDNA overhangs. In archaea, this process is mediated by the previously mentioned systems: the helicase MlaA/HerA, the MR complex, and the NurA nuclease [174, 175]. These overhangs are protected by single-stranded DNA binding proteins [50, 173, 176], an important process to prevent degradation of these ssDNA ends, as well as keeping them from base pairing which may inhibit recombination processes. The DNA binding domains of these molecules are composed of oligosaccharide/oligonucleotide binding (OB) folds. However, depending on the domain of life and further divisions there are differences in overall architecture and quaternary structures, and different naming systems are used. The human single-stranded DNA binding protein is termed replication protein A (RPA), where the protein exists as a heterotrimer. The first crystal structure of the protein revealed how the largest subunit RPA70 binds ssDNA via two tandem OB folds (Figure 6(a)) with sidechains stacking with the ssDNA bases to produce an irregularly shaped ssDNA chain [177]. The contorsion of the ssDNA is such that in the larger more intact RPA ssDNA complex structure, the bases are protected by generally facing inward [178]. In contrast, the domain organization of the bacterial single-stranded DNA binding protein (SSB) from *E. coli* consists of a heterotetramer, and while the individual domain structures contain OB-folds, they differ from eukaryotic RPA OB-folds (Figure 6(c)) [179–181].

Interestingly, archaea appear to have perhaps followed two paths for their ssDNA binding proteins. The euryarchaea subdivision contain more eukaryotic-like RPA proteins, whereas the crenarchaea subdivision tend to have ssDNA binding proteins that resemble bacterial SSBs in terms of having a more similar domain organization [182, 183]. Overall, the OB folds of both euryarchaeal RPAs and crenarchaeal SSBs resemble those of eukarya (Figure 6) [184]. However, the monomeric crenarchaeal SSB has an unconserved C-terminal extension reminiscent of bacterial proteins, where their C-terminal ends are involved with interactions with the exonuclease1 protein [184, 185]. The crenarchaeal SSB eventually led to the discovery of new eukaryotic ssDNA binding proteins, where in humans these have been termed hSSB1 and hSSB2. Initial characterization of hSSB1 led to the findings that it appeared to accumulate within the nucleus to form foci with other proteins following the induction of DSBs, where the colocalization does not seem to correlate with RPA binding at the same sites [186]. Moreover, experimental results suggested a role in HR with interactions with Rad51. Later the hSSB1 and hSSB2 proteins were found to coalesce with the INTS3 and C9orf80 proteins to form the sensor of single-stranded DNA complex 1 (SOSS1). This complex is under very active investigation and results suggest involvement with a variety of DNA damage response proteins, such as ATM, Rad51, and Exo1, through recruitment interactions, signaling, or regulation [187–189].

### 3.7. Infidelity and Fidelity

In the next step of homologous recombination the ssDNA binding proteins are dislodged and replaced by the central DNA strand exchange enzyme RadA (or Rad51) with the aid of mediators [25, 50, 190, 191]. Interestingly, eukaryotic mediator BRCA2 appears to use mimicry to accomplish this task as it also contains OB folds [50, 192]. The DNA bound RadA/Rad51 subunits form a nucleoprotein filament that invades a homologous segment of dsDNA, which then serves as a template for new DNA synthesis by polymerases such as pol D [193]. Following synthesis, the resulting Holliday junction DNA structures generated during recombination [173] are then rearranged by other enzymes known as resolvases. In the archaea, the Holliday junction cleavage (Hjc) and Holliday junction endonuclease (Hje) are examples of proteins that are implicated for this role [194–196].

The function of the central homologous pairing and strand exchange enzyme is conserved among the 3 domains of life. In the 1960s, the finding that bacterial resistance to radiation was correlated to the *recA* gene [197] was the first step to determine that the RecA protein performed the pairing and strand exchange function. While its archaeal (RadA/Rad51) and eukaryotic (Rad51) counterparts share the same function, their sequences were found to differ significantly. Archaeal Rad51/RadA proteins [198] generally have approximately 40% primary sequence identity with eukaryotic Rad51, and these enzymes also share similar overall domain architecture. In contrast, only about 20% sequence identity is shared between Rad51/RadA proteins and bacterial RecA proteins, and this is localized to a single domain. Early sequence alignment programs were unable to correctly align structurally similar regions of the bacterial RecA proteins with their functional equivalents from archaea and eukarya. ApoRecA usually exists as a protein filament [199, 200], while apoRad51 exists primarily as a polymeric ring. Similar to RadA/Rad51, in the presence of DNA, RecA will form a nucleoprotein filament [201–203].

**Figure 7:**
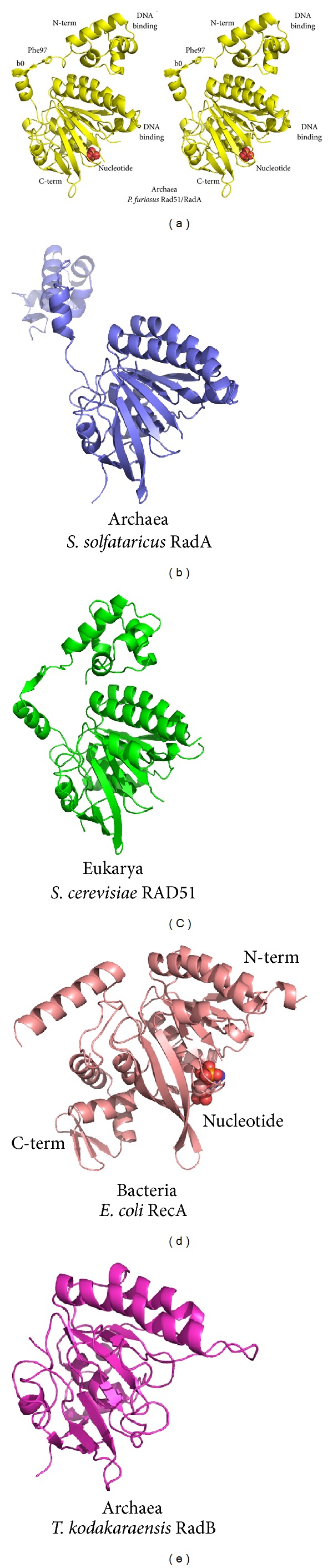
Structural comparisons of Rad51 family proteins. (a) Stereoview of archaeal *P. furiosus* Rad51 (PDB code 1PZN). While Rad51 generally forms larger homopolymeric assemblies, the prototypical fold of a single archaeal Rad51 or RadA protein consists of a small 4-helix bundle N-terminal DNA-binding domain, which is tethered to a larger C-terminal ATPase domain. The ATPase domain has several loops that are also implicated in binding DNA. The interdomain linker that tethers the two domains contains a polymerization motif (PM) that consists of a *β*-strand *β*0, which, upon contact with a neighboring subunit, extends the *β*-sheet of the ATPase domain. Conserved Phe97 forms a ball and socket to stabilize this interaction. (b) The two domains of the archaeal *S. solfataricus *structure individually superpose well with the PfRad51 domains. However, this structure reveals the flexibility of the interdomain linker, where the N-terminal domain has swung outward (PDB code 2BKE). (c) The structure of *S. cerevisiae* RAD51 reveals structural conservation with the archaeal proteins (PDB code 1SZP). (d) The bacterial RecA protein shares the ATPase domain fold (PDB code 2REB). However, in this structure the ATPase is represented in the N-terminal domain, while in archaea and eukarya the ATPase is represented in the C-terminal domain. A small N-terminal arm extends from the bacterial ATPase and serves a similar function as the Rad51 PM. (e) The archaeal *T. kodakaraensis* RadB protein structure illuminates how extensions to the ATPase likely served as critical components of primordial recombination structures, where in the archaea and eukarya the N-terminal domain became an accessory domain, whereas in bacteria the C-terminus gave rise to an accessory domain (PDB code 2CVF).

The first full-length RadA/Rad51 crystal structure solved was derived from the archaeal thermophile *P. furiosus* (PfRad51) (Figure 7(a)) [25]. A single subunit of Rad51 consists of a small N-terminal 4-helix bundle and a larger C-terminal ATPase domain. The N-terminal domain contains an HhH motif [204], which acts in Mg-coordinated DNA phosphate backbone binding [205]. The larger ATPase domain consists of a central beta-sheet surrounded between alpha-helices. The ATPase contains the Walker A and B motifs and a rare *cis*-linked glycine at position 141 within the active site. Two loops, termed L1 and L2, that are analogous to disordered loop regions found within the first RecA structure [200] and implicated to become ordered upon DNA binding, are also contained in the C-terminal ATPase domain. Comparisons with other archaeal crystal structures that followed, such as those from *Methanococcus voltae* (MvRadA) [206] and *S. solfataricus *(SsRadA) [207], revealed that the N- and C-terminal domains are highly conserved (Figure 7(b)).

**Figure 8:**
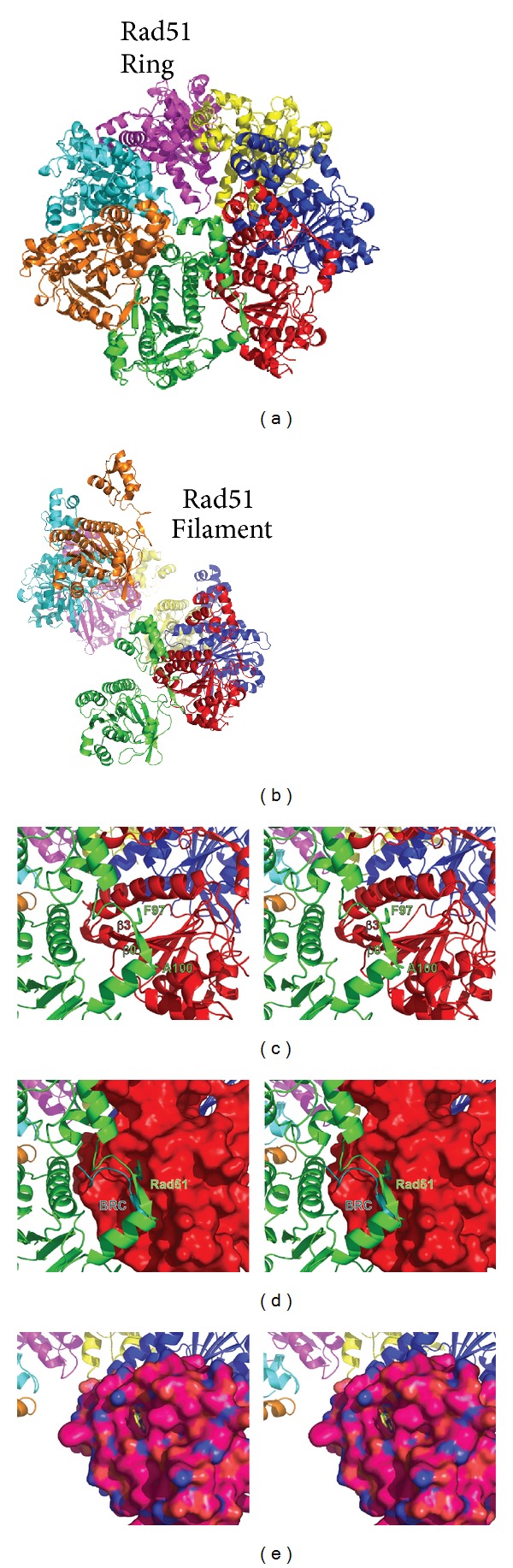
Rad51 assemblies and interface mimicry and exchange. (a) A Rad51 ring model derived from a *P. furiosus* crystal structure is composed of 7 identical subunits, each colored differently (PDB code 1PZN). While substantial intersubunit contacts are made on both sides of each individual subunit, the interactions made by the polymerization motif (PM) shown in (c) are responsible for the ability of the assembly to transition from a ring to a filament, as shown in (b). (b) A Rad51 filament model derived from docking in a *P. furiosus* crystal structure into *S. solfataricus* Rad51 electron microscopy 3D reconstruction density. This assembly generally forms upon binding DNA to generate a nucleoprotein filament, where DNA resides in the interior of the assembly. (c) Wall-eyed-stereoview zoom of Rad51 subunits from (a). The PM resides in the interdomain linker that tethers the N-terminal and C-terminal domains together. The PM *β*0 (green) extends the *β*-sheet made by the adjacent subunit (red) by bonding to *β*3. The conserved Phe buries itself into a pocket formed by the adjacent subunit. The conserved Ala residue also stabilizes the PM via hydrophobic interactions. (d) Same view as in (c), however the surface of the red subunit is shown. Overlay of the HsRad51 ATPase:BRC4 domain fusion structure (PDB code 1N0W) with PfRad51 reveals that BRC4 mimics interactions made by the Rad51 PM, and it is proposed that the interfaces may be exchanged to form Rad51:BRCA2 complexes in eukarya. (e) The same PM interface is now a target for small-molecule fragment-based approaches to develop ligands that work in conjunction with radiation and genotoxic drugs used to treat cancer. Due to the conservation of the *P. furiosus* and human enzymes, researchers developed a humanized PfRad51 mutant (overlaid in the position of the red subunit in other panels) and identified compounds that bind to the hydrophobic pocket that is normally occupied by the PM Phe residue (PDB codes 4B33, 4B34, and 4B3C). After optimization of a designed ligand, the interaction between Rad51 and BRCA2 may be prevented.

Likely the most unusual feature of the structure is the interdomain linker. This 19 amino acid linker between the N- and C-terminal domains appears to be highly flexible. In the PfRad51 structure the majority of N-terminal domains were disordered presumably due to motion and lack of crystal contacts, whereas solution SAXS measurements supported their presence. The quaternary structure of the PfRad51 consists of two oppositely stacked heptameric rings. The interdomain linker contains a polymerization motif (Rad51-PM) that consists of the conserved sequence G*FxxAxE (* = possible insertion, x = differing residues) and provides a key interface for the ring assembly [25, 50] (Figures 7(a), 8(a), and 8(c)). Residues of this motif form a beta-strand *β*0 that extends the central beta-sheet of the neighboring subunit. For additional stability, the Phe residue also buries itself into a hydrophobic pocket formed by the adjacent subunit. These interactions allow the ring assembly to transition to a helical filament during DNA binding. Visualizing the interdomain linker in subunits from the different quaternary assemblies found within the PfRad51 (ring) and SsRadA (extended structure) crystal structures illustrates the flexibility of the linker (Figures 7(a) and 7(b)). Additionally, by also comparing these structures with the MvRadA (filament), it is also revealed that the *α*5/*β*0 elbow-like bend usually remains rigid within the linker [206].

During HR, Rad51 binds to DNA in two steps, first binding to the 3′ ssDNA overhang of a DSB via the primary DNA binding site and then binding to homologous dsDNA at a secondary site. Electron microscopy (EM) studies show that the primary Rad51 DNA binding site likely lies at the center of the Rad51 filament, as for RecA [202]. DNA-bound RecA and Rad51 filaments have a large outer groove with one smooth side and one lobed side. Biochemical evidence indicates that the stoichiometry of the Rad51 nucleoprotein filament is 1 Rad51 monomer per 3 or 4 nucleotides [23, 25, 191, 208–210]. These filaments expand or contract in the presence of ATP, ADP, or other nucleotide analogs. The lobes within the DNA binding groove are likely to include the N- or C-terminal domains of Rad51 and RecA, respectively, and these regions also undergo significant nucleotide-induced conformational change [202, 203]. Filament expansion likely involves a change in DNA base rotamer conformation, as observed for RecA-bound DNA by NMR [211]. *In vitro*, Rad51 filaments bind ssDNA or dsDNA in the primary DNA binding site.

Comparing the individual subunits from the 3 domains of life, it is readily apparent that archaeal RadA/Rad51 proteins are structurally similar to eukaryotic Rad51 proteins. Despite that the subunit structures of PfRad51 and *S. cerevisiae* RAD51 (ScRAD51) [23] in Figure 7 are derived from ring and filament assemblies, respectively, they are obviously quite similar in tertiary structure. Moreover, the eukaryotic proteins also possess the Rad51-PM and have also been found to exist as rings [212] or filaments [23]. On the other hand, a structure of *E. coli* RecA (EcRecA) [200] illustrates a much different prototypical subunit organization for the bacterial DNA strand exchange proteins (Figure 7(d)). For these proteins, the ATPase domain now represents the N-terminal domain, and the C-terminus consists of a small DNA binding domain that is roughly the same size as RadA/Rad51 N-terminal domains. A small N-terminal arm consisting of an alpha-helix and linker provides the interactions for polymerization. Interestingly, many archaea contain paralogs of RadA, which could perhaps perform similar roles to eukaryal Rad51 homologs [173, 213]. Some euryarchaea contain a paralog implicated in HR that is smaller than RadA called RadB [191, 213]. This protein consists of just the ATPase domain [214] (Figure 7(e)). RadB has DNA binding properties and, depending on the species from which it was characterized, has been implicated in a variety of interactions and functions. These include inhibiting and activating DNA strand exchange [173], and interacting with RadA/Rad51, the polD polymerase, and the Hjc holiday junction cleavage protein [191, 193, 214].

### 3.8. Utility

The properties of archaeal DNA repair enzymes, the similarity to eukaryotic homologs, and stability, are being exploited for inhibitor design. In eukaryotes, unrepaired DNA double-strand breaks (DSBs) can trigger cells to undergo programmed cell death, a process known as apoptosis. Alternatively, DSBs may lead to gross chromosomal rearrangements or loss, thus threatening genome stability. Illustrating the importance of Rad51 in metazoans, knockout mice deficient in Rad51 die during embryogenesis [215], and chicken DT40 cells lacking Rad51 show reduced viability [216]. The breast cancer susceptibility protein BRCA2 acts as a mediator for generating Rad51 nucleoprotein filaments, thus playing a role in HR. Women who carry a BRCA2 mutation have a greatly increased lifetime risk for developing breast or ovarian cancer [217]. The central region of BRCA2 contains a set of 8 noncontiguous ~30 amino acid repeat sequences. These sequences termed BRC repeats contain many tumorigenic polymorphisms, where a single mutation within a repeat can increase cancer risk [24, 218]. BRC repeats bind directly to the Rad51 filament to mediate their loading onto DNA; however BRC repeat-derived peptides prevent Rad51 polymerization into rings and nucleoprotein filaments *in vitro *[219, 220] and prevent nuclear aggregates of Rad51 *in vivo *[221]. By overlaying the PfRad51 structure with a structure of the human Rad51 ATPase domain fused to repeat BRC4 [24], it was revealed that the BRC repeat-derived peptide mimics the Rad51-PM and would disrupt Rad51:Rad51 intersubunit interactions, so that they may be loaded onto DNA as individual subunits (Figure 8) [25, 50]. While BRC repeats do not bind archaeal RadA/Rad51 proteins, the extreme structural similarity between PfRad51 and HsRad51 allowed the generation of a mutant PfRad51 that could be bound by BRCA2 and transported to nuclei in irradiated human cells that would contain DSBs. The further utility of the archaeal PfRad51 enzyme is being exploited as a platform for drug design (Figure 8(e)), as it is more stable and homogeneous in solution than the human enzyme [26]. Again, a few mutations replicate the surface properties of the human enzyme at the pharmaceutical target site, the binding site of the Rad51-PM.

## 4. Conclusions and Prospects

With the first views of cells under the light microscope, classifying microbes without nuclei together against cells from animals and plants with nuclei was indeed intuitive. With the advent of techniques such as X-ray crystallography and NMR, a closer look “under the hood” revealed that some of the “parts” of these cells certainly resembled those believed to be parts of more distant relatives, paralleling insights from available sequencing data. The stability of macromolecules from archaeal thermophiles often allows obtaining “the first structure” of a class of enzymes. Furthermore, the realization that the overall folds and architectures between many archaeal and eukaryal proteins are similar is a huge benefit for using structures to understand human disease. For instance, not only are the tertiary structures often conserved, but also at the primary level residues that result in disease when mutated are often conserved. This aids interpretation of mechanistic defects at the basic research level and the use of structures at the application level. As systems that inform responses to extreme environmental stress, the archaeal proteins provide biological insights along with precise structural knowledge of complexes and conformations that are often prototypical and foundational. Defining the abilities and limits of the adaptive strategies employed by extremophiles to thrive under extreme stress is also relevant to determining the chemical and physical boundaries that limit life on Earth and beyond for life as we know it. The huge efforts on human systems often provide complementary information so that the combination of archaeal and human structural and biological data provides a deep and comprehensive understanding of great value and utility.

Here we highlighted such prototypic examples of DNA replication and repair systems, and, for several of these proteins, archaeal structures predated those of human structures or served as the only representatives of their class of protein. We have noted that archaea provide deep insights into mechanisms of maintaining genome integrity in the face of extreme environmental stress, with prospects of temperature-trapping flexible complexes and revealing core domains and transient and dynamic complexes. Indeed, archaeal windows into genome integrity have proven exceptionally bright and clear compared to other choices. In concert with archaeal systems, bacterial thermophiles such as *T. maritima* have also provided pertinent examples for X-ray crystallographic studies of proteins involved in DNA damage responses but are outside the scope of this review. However, these include recombination repair by RuvB [222], nucleotide excision repair by UvrC [223], and deaminated base excision repair by endonuclease V [224]. Likewise, many informative archaeal topoisomerase structures have added greatly to our understanding of these systems [225, 226], but these examples came after other human and *E. coli* topoisomerase family structures [227–230] and are also outside the scope of this review, which again is mainly focused upon systems where archaeal results had led the way to understanding human systems and processes.

Archaeal proteins have allowed us and other researchers to start the process of bridging structures to pathways and systems. They can provide structures that are not just “parts-lists” but include interactions and conformations that link to functional networks. The coupling of advanced archaeal genetics and advanced structural methods to combine MX and SAXS promises to provide an integrated and predictive knowledge of the dynamic structural machines critical to cell biology [231, 232]. For instance, the development of genetics for prototypic archaeal systems such as *Sulfolobus* [233, 234] and *Pyrococcus* [67, 235, 236] coupled to advanced small-angle X-ray scattering methods [237–239] are but a few powerful applications of these advances. Although many archaea are anaerobic, the development of anaerobic iLOV as well as the aerobic green fluorescent protein GFP now allows fluorescent labeling of archaeal proteins in anaerobic and aerobic systems [240, 241]. Going forward, a structural and mechanistic understanding of critical networks, such as those that respond to environmental stress and change, will enable applications such as rewiring bugs for synthetic biology and biomanufacturing. Archaea furthermore provide insights into responses to environmental stresses, such as heavy metal ions, that pose challenges for DNA integrity and repair [242]. Currently, archaeal DNA enzymes are already widely used in biotechnology for PCR [243] and detection assays [244]. Furthermore experiments with SAXS show archaeal thermophiles allow the direct testing and visualization of dynamic ATP-driven conformational changes that control different biological outcomes [245]. Thus, we can expect archaeal structural biology to remain both important and vibrant in the next decade with both medical and industrial impacts.
